# CMP-Neu5Ac Hydroxylase Null Mice as a Model for Studying Metabolic Disorders Caused by the Evolutionary Loss of Neu5Gc in Humans

**DOI:** 10.1155/2015/830315

**Published:** 2015-10-19

**Authors:** Deug-Nam Kwon, Yun-Jung Choi, Ssang-Goo Cho, Chankyu Park, Han Geuk Seo, Hyuk Song, Jin-Hoi Kim

**Affiliations:** Department of Animal Biotechnology, Konkuk University, Seoul 143-701, Republic of Korea

## Abstract

The purpose of this study was to identify the modification/turnover of gene products that are altered in humans due to evolutionary loss of Neu5Gc. CMP-Neu5Ac hydroxylase- (*Cmah*-) deficient mice show the infiltration of Kupffer cells within liver sinusoids, whereas body and liver weight develop normally. Pathway analysis by use of Illumina MouseRef-8 v2 Expression BeadChip provided evidence that a number of biological pathways, including the glycolysis, gluconeogenesis, TCA cycle, and pentose phosphate pathways, as well as glycogen metabolism-related gene expression, were significantly upregulated in *Cmah*-null mice. The intracellular glucose supply in *Cmah*-null mice resulted in mitochondrial dysfunction, oxidative stress, and the advanced glycation end products accumulation that could further induce oxidative stress. Finally, low *sirtuin-1* and *sirtuin-3* gene expressions due to higher NADH/NAD in *Cmah*-null mice decreased *Foxo-1* and *MnSOD* gene expression, suggesting that oxidative stress may result in mitochondrial dysfunction in *Cmah*-null mouse. The present study suggests that mice with CMAH deficiency can be taken as an important model for studying metabolic disorders in humans.

## 1. Introduction

The CMP-Neu5Ac hydroxylase (*Cmah*) gene contains the same mutation in every person studied to date [1, 2]. This implies that all humans inherited the mutation from a common ancestor. Loss of* N*-glycolylneuraminic acid (Neu5Gc) greatly increases the levels of* N*-acetylneuraminic acid (Neu5Ac) in human cells [3]. Microbes or viruses approaching the surface of a human cell are likely to first encounter members of a family of sugars known as sialic acids that include Neu5Ac and Neu5Gc, which show specificity for the particular structure and linkage of sialic acid targets [4]. Thus, loss of Neu5Gc in humans has imparted protection against some animal pathogens, whereas preferential binding to Neu5Ac can enhance the success of other pathogens. Regardless of the mechanism by which Neu5Gc is lost, the question arises, whether the loss of Neu5Gc in humans has had some beneficial effects during the course of human evolution. If it did not, the question of the side effects of its loss remains. Deng et al. [5] reported that typhoid toxin binds and is toxic to cells and mice constitutively expressing* Cmah* are resistant to typhoid toxin. It has been reported that some animal forms of influenza A virus bind to Neu5Gc preferentially, whereas human forms show some preference for Neu5Ac [6]. The merozoite form of* Plasmodium falciparum* invading red blood cells uses cell surface Neu5Ac as targets for initial binding [7–9]. Martin et al. [10] reported that differences in human and chimpanzee susceptibility to malaria were related to the genetic loss of Neu5Gc in humans, and Löfling et al. [11] suggested that canine and feline parvoviruses preferentially recognize the nonhuman cell surface sialic acid Neu5Gc. Springer et al. [12] reported the presence of human-like sialic acid in the new world monkeys, comprising a third of all primate species.

Using a knockout technique,* Cmah* has been deactivated in mice [13] and pigs [14], and the effects have been observed in vivo. The resulting mice showed Neu5Ac accumulation similar to that observed in humans, as well as the appearance of associated problems that are common in humans: diminished acoustic sensitivity, reduced startle response threshold (relative to normal mice), hearing loss in old age, delayed skin healing in adulthood, and a human-like muscular dystrophy phenotype that results from a combined mutation of the dystrophin gene [15, 16]. Immunologically, these mice can show enhanced B cell proliferation and antibody production, an association supported by a previous study showing that Neu5Gc is physiologically downregulated during normal B cell activation [13]. Very recently, we also reported that Neu5Gc loss in the heart, lung, and kidney of* Cmah*-null mice affected the complex regulation of cellular signaling pathways known to be involved in human diseases [17].

Recently, Kavaler et al. [18] showed that high fat diet- (HFD-) fed* Cmah*-null mice exhibit fasting hyperglycemia and glucose intolerance, as well as dysfunction of pancreatic *β*-cell in type 2 diabetes. This same report demonstrated that sialylation with Neu5Gc plays an important role in insulin-producing *β*-cell function and that its loss contributes to *β*-cell dysfunction in mice. However, the mechanisms of impaired glucose tolerance and *β*-cell dysfunction arise in HFD-fed* Cmah*-null mice which remain unknown. To elucidate this previously observed phenotype of* Cmah*-null mice, we recently reported that microRNA (miRNA) dysregulation in* Cmah*-null mouse-derived liver cells could impair insulin/PI3K-AKT expression signaling during the transition from glucose tolerance to intolerance [19].

Despite growing interest in the relationship between* Cmah* and systemic metabolism, how genome-wide alterations in gene expression in the livers of* Cmah*-null mouse affect metabolism remains unclear. The present study used differential expression profiling of livers from control and* Cmah*-null mice using Illumina MouseRef-8 v2 Expression BeadChip for whole-genome analysis. We further validated the expression of selected genes by quantitative real-time reverse transcriptase polymerase chain reaction (RT-qPCR) analysis or pathway-focused glucose metabolism PCR array and/or microRNA PCR array analyses.

## 2. Materials and Methods

### 2.1. Animal Ethics

All mouse lines were maintained on a congenic C57Bl/6J background. Mice were permitted to eat and drink ad libitum and were fed standard mouse chow (Cargill Agri Purina, Inc., Seongnam-Si, Korea). Twelve-week-old wild type (WT) and* Cmah*-null male mice were used. This study was carried out in strict accordance with the recommendations in the Guide for Care and Use of the Konkuk University Animal Care and Experimentation Community. The study protocol was approved by the Committee on the Ethics of Animal Experiments of Konkuk University (IACUC approval number KU12045).* Cmah* <tm1Ykoz> null mice were kindly provided by RIKEN Center for Developmental Biology (RIKEN CDB, Japan).

### 2.2. Genotyping of* Cmah*-Null Mice

Genomic DNA was isolated from the tails of* Cmah*-null mice using a DNeasy Blood and Tissue kit, according to the manufacturer's protocol (Qiagen, Valencia, CA, USA). The conditions for PCR amplification were as follows: an initial denaturation for 3 min at 95°C, followed by 30 cycles of 30 sec at 94°C for denaturation, 30 sec at 58°C for annealing, and 30 sec at 72°C for extension. PCR products were analyzed on 1.2% agarose gels.

### 2.3. Immunohistochemistry (IHC) and Immunofluorescence Staining

For the IHC analyses, the tissues were fixed in 10% neutral buffered formalin and then embedded in slides. After blocking with Background Sniper solution, the tissue sections were incubated with selective primary antibodies for CMAH (Santa Cruz, Texas, USA; 1 : 100) and Neu5Gc (Sialix, San Diego, CA, USA; 1 : 200) at 4°C overnight. Following incubation, the samples were washed and incubated again with horseradish peroxidase-conjugated secondary antibody. Samples were then stained with ImmPACT DAB peroxidase substrate (Vector Laboratories, CA, USA) to visualize the signal. After staining with Hematoxylin QS, the samples were mounted using VECTASHIELD HardSet mounting medium (Vector Laboratories) and observed using fluorescence microscopy (Olympus, Japan). For immunofluorescence analysis, the samples were incubated at 4°C overnight with specific primary antibodies to F4/80 (Abcam, Cambridge, UK; 1 : 100) and mitochondria (Abcam; 1 : 100), followed by incubation with Alexa Fluor 488-labeled goat anti-rat antibody (1 : 200) and Alexa Fluor 568-labeled goat anti-mouse antibody (1 : 300) (Supplementary Table  1 in Supplementary Material available online at http://dx.doi.org/10.1155/2015/830315). The samples were mounted using VECTASHIELD HardSet mounting medium with DAPI (Vector Laboratories) and observed using fluorescence microscopy (Olympus, Japan).

### 2.4. Microarray Analysis

#### 2.4.1. RNA Preparation

Total RNA was extracted and purified from the livers of WT and* Cmah*-null mice using an RNeasy Mini Kit (Qiagen) according to the manufacturer's protocol. For quantitation and quality checking, samples were analyzed on an Agilent 2100 Bioanalyzer (Agilent Technologies, Palo Alto, USA).

#### 2.4.2. Labeling and Purification

Total RNA was both amplified and purified by the Ambion Illumina RNA amplification kit (Ambion, Austin, USA) according to the manufacturer's instructions. After purification, the cRNA was quantified using a ND-1000 Spectrophotometer (NanoDrop, Wilmington, USA).

#### 2.4.3. Hybridization and Data Export

Labeled cRNA samples (750 ng) were hybridized to each mouse-8 expression bead array for 16–18 h at 58°C, following the manufacturer's instructions (Illumina, Inc., San Diego, USA). Array signals were detected using Amersham fluorolink streptavidin-Cy3 (GE Healthcare Bio-Sciences, Little Chalfont, UK), according to the manual supplied with the bead array. Arrays were then scanned with the help of an Illumina BeadArray Reader confocal scanner, following the manufacturer's instructions.

#### 2.4.4. Raw Data Preparation and Statistical Analysis

Both the quality of hybridization and overall chip performance were examined by visual inspection of the internal quality control checks as well as raw scanned data. Raw data was extracted by the software provided by the manufacturer (Illumina GenomeStudio v2009.2 [Gene Expression Module v1.5.4]). Array data were filtered for a *p* value < 0.05 in at least 50% of samples. Signal values from the selected genes were log-transformed and normalized using the quantile method. WT and* Cmah*-null mice groups were comparatively analyzed for differences in fold change. Gene ontology (GO) analysis of the probe signals that differed significantly between the two groups was performed using PANTHER (http://www.pantherdb.org/panther/ontologies.jsp). All data analyses and visualization of differentially expressed genes were performed using the R 2.4.1 statistical package (https://www.r-project.org/).

### 2.5. Pathway-Focused PCR Array Analysis

Changes in gene expression were analyzed using a pathway-focused glucose metabolism PCR array (SABiosciences, Valencia, CA, USA). Total RNA was reversely transcribed using an RT^2^ First-Strand Kit (Qiagen). cDNA from individual samples was used as a template for the PCR array, according to the array instructions, using SYBR green (Bio-Rad, Hercules, CA, USA) on an ABI ViiA 7 system (Applied Biosystems). Data were analyzed using SABiosciences RT^2^ Profiler PCR Data Analysis software, available at http://pcrdataanalysis.sabiosciences.com/pcr/arrayanalysis.php, and were considered significant at >1.5-fold change. Relative quantitation of each gene was performed by normalization to four housekeeping genes (*Actb*,* B2 m*,* Gapdh*, and* Gusb*), and the WT and* Cmah*-null groups were compared using the 2^−ΔΔCt^ method.

### 2.6. miRNA Isolation and cDNA Generation

miRNA was isolated from mouse tissue using the miRNeasy Mini Kit (Qiagen), following the manufacturer's instructions. The concentration and purity of the isolated miRNA were measured using a NanoDrop 2000 spectrometer. Conversion of miRNA to cDNA was performed using 100 ng of miRNA, according to the manufacturer's instructions for the RT^2^ miRNA First-Strand Kit (SABiosciences, Frederick, MD).

### 2.7. MicroRNA PCR Array Analysis

Differences in miRNA expression between samples were measured using a Liver miFinder miRNA PCR array (SABiosciences, Frederick, MD). The cDNA from individual samples was used as a template for the SYBR green PCR array, according to the instructions for an ABI ViiA 7 system (Applied Biosystems). Data were analyzed using SABiosciences RT^2^ Profiler PCR Data Analysis software (available at http://pcrdataanalysis.sabiosciences.com/mirna/arrayanalysis.php) and were considered significant at >1.5-fold change. Relative quantitation of each miRNA was performed by normalizing to the small nuclear RNA housekeeping panel (*Snord61*,* Snord68*,* Snord72*,* Snord95*,* Snord96a*,* Rnu6-2*), and the WT and* Cmah*-null samples were compared using the 2^−ΔΔCt^ method.

### 2.8. RT-qPCR

The total RNA obtained from each tissue was reverse-transcribed using the QuantiTect Reverse Transcription Kit (Qiagen). To assess gene expression, RT-qPCR was conducted using an ABI ViiA 7 system (Applied Biosystems) and SYBR green (see Supplementary Tables  2, 3, and 4 for RT-qPCR primer sets).* Gapdh* was used as an internal control to normalize the RT-qPCR efficiency and to quantify gene expression in the control and* Cmah*-null liver-derived mRNA. We performed RT-qPCR on each sample independently and in triplicate.

### 2.9. Mitochondrial DNA (mtDNA) Quantification

For quantification of mtDNA, essentially the same protocol was used as for RT-qPCR, with 25 ng genomic DNA used as a template, with normalization of* CytB* amplification levels against the nuclear *β-actin* gene (Supplementary Table  2).

### 2.10. Western Blot Analysis

The tissues were sonicated in a radioimmunoprecipitation assay (RIPA) buffer (containing a protease inhibitor cocktail) and supernatants were transferred after centrifugation. The protein concentration of the samples was measured by a Bradford assay kit (Bio-Rad), using bovine serum albumin as a standard. Proteins were electrophoresed on 10% SDS-PAGE gels and subsequently transferred to PVDF membranes. Blots were incubated with specific primary antibodies for CMAH (Santa Cruz), Neu5Gc (Sialix), USP2 (Proteintech, IL, USA), and GCK (Santa Cruz), followed by a horseradish peroxidase- (HRP-) conjugated secondary antibody (Calbiochem, CA, USA), and developed using the ECL detection system (Amersham Pharmacia Biotech Inc., NJ, USA) (Supplementary Table  1). An anti-actin antibody was used to verify equal protein loading. Band intensities of each protein profile were quantified by image processing and analyzed using ImageJ v1.32.

### 2.11. Data Mining

Significantly affected or differentially expressed genes were subjected to intensive investigation to identify their biological functions. Gene interaction networks, functions, and pathways analyses were created by Ingenuity Pathway Analysis (IPA; Ingenuity Systems, Mountain View, CA, USA), which assists in microarray data interpretation via grouping differentially expressed genes (DEG) into known functions, pathways, and networks, based primarily on human and rodent studies. The recognized genes were then mapped to genetic networks that were available in the Ingenuity Database and then ranked by score. Significance was set at a *p* value of 0.05. Data were also analyzed using the Kyoto Encyclopedia of Genes and Genomes (KEGG) database (http://www.genome.jp/kegg/) to identify their biological functions. In addition, DAVID Functional Annotation tools (http://david.abcc.ncifcrf.gov/) were utilized to study the biological function of the differentially regulated genes [20].

### 2.12. Statistical Analysis

All experimental data are presented as means ± standard deviation (SD). Each experiment was performed at least three times. For statistical analysis (Figures 5 and 6), one-way analysis of variance (ANOVA) was performed to determine whether there were differences among the groups, and Fisher's posttest was performed to determine significance between pairs of groups. In all experiments, ^*∗*^ *p* < 0.05, ^*∗∗*^ *p* < 0.01, and ^*∗∗∗*^ *p* < 0.001 were considered significant.

## 3. Results

### 3.1. CMAH Deficiency Results in Infiltration of Kupffer Cells within Liver Sinusoids, but Body and Liver Weight Develop Normally

Kupffer cells, tissue macrophages residing within the lumen of the liver sinusoids, are considered to play a key role in modulating the inflammation observed in most experimental models of liver injury [21, 22]. In order to determine the role of Kupffer cells in* Cmah*-deficient mice,* Cmah*-null mice were obtained as described previously [15, 16]. The gene knockout in* Cmah*-null mice was confirmed by PCR (Supplementary Figure 1(A)), and the loss of CMAH and its product, Neu5Gc protein expression, was confirmed by western blotting (Supplementary Figure  1(B)). To minimize differences in age and time of sacrifice, which could affect metabolism, all animals were sacrificed at zeitgeber time 9 (3 h before lights off). Liver tissues were extracted from 12-week-old* Cmah*-null and control mice (*n* = 6), and then transcriptional profiling was performed using microarray or immunohistochemical analyses. Immunohistochemical analysis showed that control mice expressed both CMAH and Neu5Gc epitopes, whereas* Cmah*-null mice showed a complete deficiency of both CMAH and Neu5Gc epitopes in liver tissues (Supplementary Figure  1(C)).

**Figure 1:**
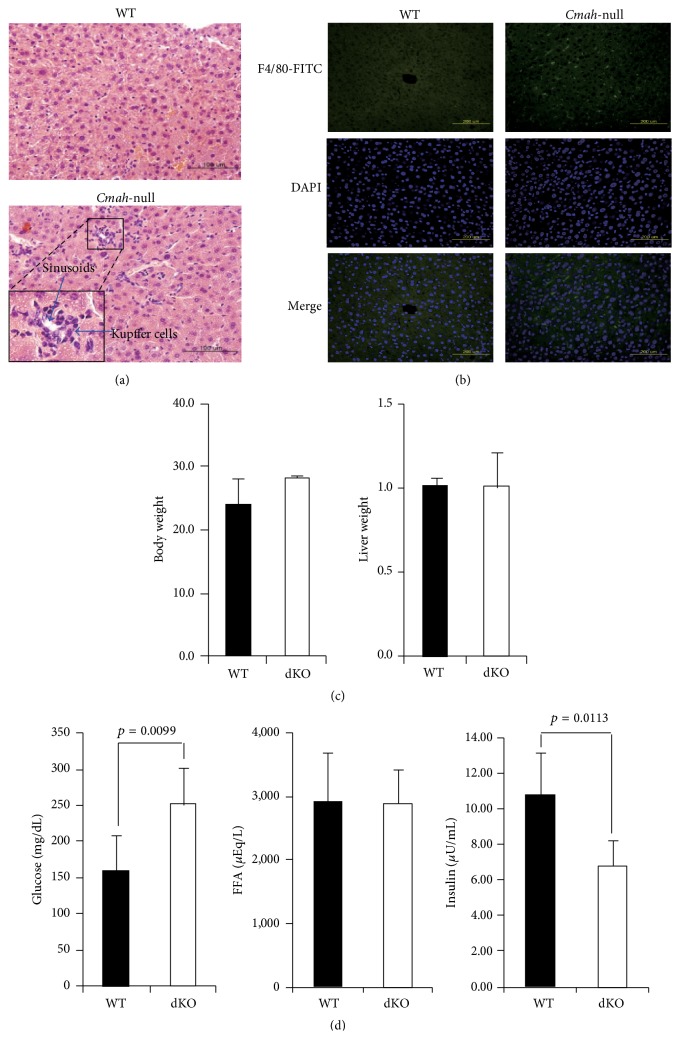
*Cmah*-null mice show impaired insulin secretion in response to glucose. (a) Liver abnormality in* Cmah*-null mice. The number of Kupffer cells was significantly higher in* Cmah*-null mouse-derived liver tissues than in WT. Bar: 100 *μ*m. (b) Immunofluorescence staining of* Cmah*-null-derived liver tissue to detect expression of Kupffer cells using F4/80 antibody. (c) Body and liver weights of WT and* Cmah*-null mice. (d) Quantity of glucose (mL/dL), FFA (*μ*Eq/L), and insulin (*μ*U/mL) in sera of WT and* Cmah*-null mice. Significant differences are indicated by *p* value.

Gross and microscopic analyses of* Cmah*-null mouse-derived liver tissues revealed abundant infiltration of Kupffer cells, which reside within the lumen of the liver sinusoids, at 12 weeks (Figure 1(a)). Since F4/80 antigen is expressed by the majority of mature macrophages, including liver Kupffer cells, we used an anti-mouse F4/80 antigen antibody to further examine the presence of Kupffer cells. As shown in Figure 1(b), F4/80 antigen antibody-positive cells were not observed in WT-derived liver, whereas* Cmah*-null mouse-derived livers showed the presence of many F4/80 antigen antibody-positive cells.* Cmah*+/−  ×* Cmah*+/− crosses showed there is no significant difference from wild type. In addition, the body and liver weights of* Cmah*-null mice that fed on standard mouse chow were not different from those of WT mice (Figure 1(c)). Even though the free fatty acid (FFA) concentration was not altered, the glucose concentration was markedly higher and the insulin concentration was significantly lower in* Cmah*-null mice than in control mice (Figure 1(d)).

### 3.2. Functional Categories of Genes with Altered Expression

**Figure 2:**
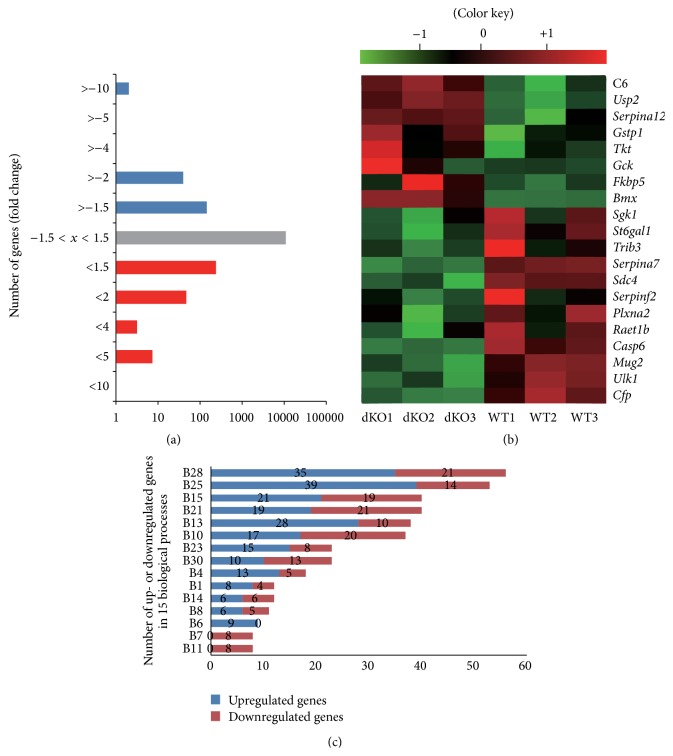
Alteration of gene expression in liver tissues of* Cmah*-null mice. (a) Histogram showing genes with a >1.5-fold difference in expression levels in liver tissues of* Cmah*-null mice. Blue and red bars indicate down- and upregulated genes, respectively. (b) Cluster analysis of 20 microarray pieces of data for WT and* Cmah*-null mice. (c) The differentially up- or downregulated genes were examined according to biological process. B1: amino acid metabolism, B2: apoptosis, B3: blood circulation and gas exchange, B4: carbohydrate metabolism, B5: cell adhesion, B6: cell cycle, B7: cell proliferation and differentiation, B8: cell structure and motility, B9: coenzyme and prosthetic group metabolism, B10: developmental processes, B11: electron transport, B12: homeostasis, B13: immunity and defense, B14: intracellular protein traffic, B15: lipid, fatty acid, and steroid metabolism, B16: miscellaneous, B17: muscle contraction, B18: neuronal activities, B19: nitrogen metabolism, B20: nonvertebrate processes, B21: nucleoside, nucleotide, and nucleic acid metabolism, B22: oncogenesis, B23: other metabolisms, B24: phosphate metabolism, B25: protein metabolism and modification, B26: protein targeting and localization, B27: sensory perception, B28: signal transduction, B29: sulfur metabolism, B30: transport, and B31: biological process unclassified. The bar graph includes up- and downregulated genes in the top 15 biological process categories.

The gene expression profiles of livers from control and* Cmah*-null mice were compared using an Illumina MouseRef-8 v2 Expression BeadChip, which is composed of 25,697 probe sets for mouse mRNA. After performing unpaired *t*-test analyses with Bonferroni correction, 366 genes, which showed a ≥1.5-fold change in expression and *p* < 0.05, were identified. Among these, 204 genes were upregulated, whereas 162 genes were downregulated in* Cmah*-null mice-derived livers compared to control mouse-derived livers. The complete list of these genes is shown in Additional Files 1 and 2. Microarray data have been provided in NCBI's Gene Expression Omnibus and are accessible through GEO Series accession number GSE59964 (gene expression profiles of* Cmah* gene depletion in mouse liver, lung, kidney, and heart). Genes whose expressions differed between groups were subjected to hierarchical clustering analysis, revealing that* Cmah-*null mouse-derived livers were distinct from control mouse-derived livers, with variation in gene expression ranging from −22.07 to 15.69 (Figure 2(a)). Supervised hierarchical clustering of these transcripts was used to distinguish between control and* Cmah-*null mouse-derived livers (Figure 2(b)).

To investigate the biological roles of genes that showed significant differences in expression in liver tissue of* Cmah*-null versus WT mice by microarray analysis, we used the PANTHER database for assignment of genes and to establish GO classifications. The PANTHER classification system indicated that the upregulated or downregulated gene expression in the livers of* Cmah*-null mice could be classified into biological groups, according to their functional properties; each gene corresponded to one or more groups on the basis of the function of its predicted protein (Figure 2(c)). In the classification based on 31 biological groups (Additional Files 3 and 4), the 15 groups that contained the most up- or downregulated genes were as follows: signal transduction (B28: 56 genes, 11.38%); protein metabolism and modification (B25: 53 genes, 10.77%); lipid, fatty acid, and steroid metabolism (B15: 40 genes, 8.13%); nucleoside, nucleotide, and nucleic acid metabolism (B21: 40 genes, 8.13%); immunity and defense (B13: 38 genes, 7.72%); developmental processes (B10: 37 genes, 7.52%); other metabolisms (B23: 23 genes, 4.67%); transport (B30: 23 genes, 4.67%); carbohydrate metabolism (B4: 18 genes, 3.66%); amino acid metabolism (B1: 12 genes, 2.44%); intracellular protein traffic (B14: 12 genes, 2.44%); cell structure and motility (B8: 11 genes, 2.24%); cell cycle (B6: 9 genes, 1.83%); cell proliferation and differentiation (B7: 8 genes, 1.63%); and electron transport (B11: 8 genes, 1.63%) (Figure 2(c)). In addition, genes found to differ significantly in their expression between* Cmah*-null and control mice, based on the results of the microarray analysis, were subjected to KEGG molecular pathway analysis and assigned to various KEGG molecular pathways using DAVID Functional Annotation Bioinformatics Microarray Analysis tools. The differentially regulated genes were found to belong to nine predicted pathways: drug metabolism, retinol metabolism, xenobiotics metabolism by cytochrome P450, arachidonic acid metabolism, steroid hormone biosynthesis, glutathione metabolism, insulin signaling pathway, complement and coagulation cascades, and fatty acid metabolism (Table 1).

### 3.3. Alteration of the Expression of Metabolism-Related Genes Caused by CMAH Deactivation in Mouse Livers

For further functional analysis of the consequences of CMAH loss, we identified three biological pathways using the KEGG and the PANTHER tools. The three groups, shown in Table 2, were as follows: protein metabolism and modification-related pathway (protein modification, protein folding, proteolysis, and protein biosynthesis); carbohydrate metabolism-related pathway (glycolysis, glycogen metabolism, other glycogen metabolisms, carbohydrate metabolism, monosaccharide metabolism, and other polysaccharide metabolisms); immunity and defense-related pathway (T-cell-mediated immunity, macrophage-mediated immunity, complement-mediated immunity, stress response, and detoxification). These data indicate that the loss of CMAH activity leads to both up- and downregulation of gene expression in liver tissues and regulates pathways related to carbohydrate metabolism.

**Figure 3:**
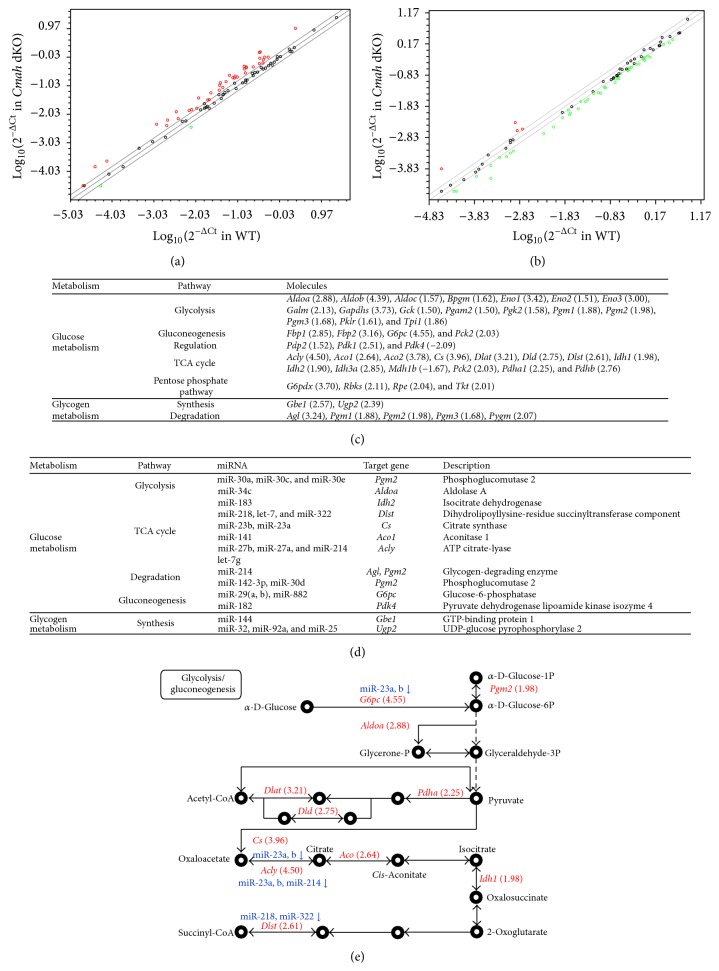
Alteration of genes and miRNAs involved in metabolism-related pathways by CMAH disruption. (a) Comparison of the relative expressions of 84 genes involved in glucose metabolism between WT and* Cmah*-null mouse-derived liver tissues using a pathway-focused glucose metabolism PCR array. The figure depicts a scatter plot of the relative expression levels. Red and green colors indicate upregulation and downregulation of gene expression (>1.5-fold change), respectively. (b) Comparison of the relative expression levels of the 84 most abundantly expressed and best characterized miRNAs in miRBase using a miFinder miScript miRNA PCR Array. The figure depicts a scatter plot of the relative expression levels. Red and green colors indicate upregulation and downregulation of miRNA expression (>1.5-fold change). (c) Metabolism-related pathways and target molecules identified in liver tissues of* Cmah*-null mice. (d) miRNAs and target genes involved in glucose and glycogen metabolism. (e) Illustration of genes involved in glucose metabolism identified by the PCR array. Red and blue colors indicate upregulated genes and downregulated miRNAs, respectively.

To clarify the altered expressions of metabolism-related genes and miRNAs resulting from CMAH inactivation, we examined the expression of genes related to this pathway in the liver of control and* Cmah*-null mice, using pathway-focused glucose metabolism PCR array (Figure 3(a)) and Liver miFinder microRNA PCR array analyses (Figure 3(b)). Expression of genes related to glycolysis [*Aldoa* (2.88),* Aldob* (4.39),* Aldoc* (1.57),* Bpgm* (1.62),* Eno1* (3.42),* Eno2* (1.51),* Eno3* (3.00),* Galm* (2.13),* Gapdhs* (3.73),* Gck* (1.50),* Pgam2* (1.50),* Pgk2* (1.58),* Pgm1* (1.88),* Pgm2* (1.98),* Pgm3* (1.68),* Pklr* (1.61), and* Tpi1* (1.86)], gluconeogenesis [*Fbp1* (2.85),* Fbp2* (3.16),* G6pc* (4.55), and* Pck2* (2.03)], the TCA cycle [*Acly* (4.50),* Aco1* (2.64),* Aco2* (3.78),* Cs* (3.96),* Dlat* (3.21),* Dld* (2.75),* Dlst* (2.61),* Idh1* (1.98),* Idh2* (1.90),* Idh3a* (2.85),* Pck2* (2.03),* Pdha1* (2.25), and* Pdhb* (2.76)], pentose phosphate pathway (PPP) [*G6pdx* (3.70),* Rbks* (2.11),* Rpe* (2.04), and* Tkt* (2.01)], and glycogen metabolism [*Gbe1* (2.57) and* Ugp2* (2.39) for synthesis and* Agl* (3.24),* Pgm1* (1.88),* Pgm2* (1.98),* Pgm3* (1.68), and* Pygm* (2.07) for degradation] was found to be significantly upregulated in* Cmah-*null mouse livers compared to controls (Figure 3(c)). In contrast, expression levels of pyruvate dehydrogenase kinase 4 (*Pdk4*) and malate dehydrogenase 1b (*Mdh1b*) were decreased −2.09- and −1.67-fold, respectively, in* Cmah*-null mice. A previous study showed that pyruvate dehydrogenase in diabetes patients is significantly downregulated [23]. In this study, we surveyed miRNAs that putatively target the glucose metabolism-related genes showing increased or decreased expression. Expression levels of five miRNAs [miR-23a (−1.52), miR-23b (−1.58), miR-34 (−1.78), miR-214 (−2.31), and miR-322 (−2.31)] were shown to be significantly decreased in* Cmah*-null mouse-derived livers by Liver miFinder microRNA PCR array analysis (Figure 3(d)). Thus, the expression levels of genes involved in glucose metabolism were increased by dysfunctional miRNAs that targeted these genes. As shown in Figure 3(e), we also identified altered expression of genes and miRNAs involved in the glucose metabolism pathway using the PCR array. Collectively, the pathway-focused glucose metabolism PCR array and Liver miFinder microRNA PCR array analyses strongly suggested that the loss of CMAH function and related evolutionary consequences make humans more prone to type 2 diabetes than other mammals.

### 3.4. Pathway Networks Derived from DNA Chip-Mapping Data

**Figure 4:**
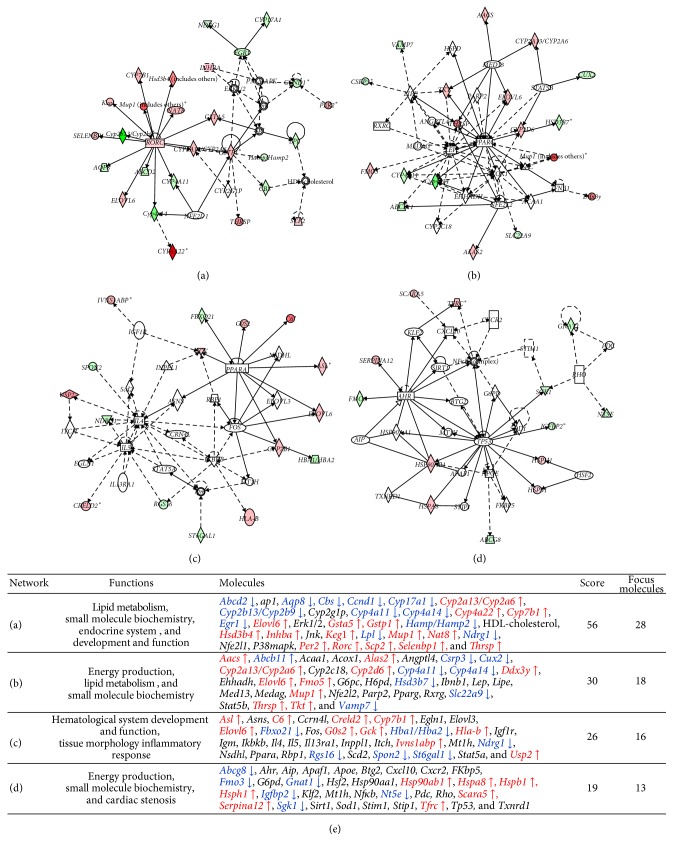
Networks predicted by Ingenuity Pathway Analysis in* Cmah*-null mice. (a) Lipid metabolism, small molecule biochemistry, and endocrine system development and function. (b) Energy production, lipid metabolism, and small molecule biochemistry. (c) Hematological system development and function, tissue morphology, and inflammatory response. (d) Cell death and survival, embryonic development, and organ development, *p* < 0.05. Red and green denote upregulation and downregulation of genes, respectively. Solid lines and dotted lines indicate direct and indirect relationships, respectively. (e) List of genes involved in networks (a)–(d). This table includes their functions, molecules, score, and focus molecules. Red and blue indicate up- and downregulated genes, respectively, which are involved in the networks.

The differential patterns of mRNA expression revealed by array analysis of* Cmah-*null mouse-derived livers were analyzed using Ingenuity System Database. In this study, we identified five networks that regulate transcription factors and pathways. The IPA of those five network-eligible identifiers revealed four statistically significant pathway networks (Figure 4).

Network pathway analysis integrated the majority of the gene expression alteration data identified by DNA chip analysis. Network 1 shows the lipid metabolism, small molecule biochemistry, endocrine system, and development and function-related signal pathways (Figure 4(a)), including 35 nodes (genes); 28 genes (*Abcd22*,* Aqp8*,* Cbs*,* Ccnd1*,* Cyp17a1*,* Cyp2a13/Cyp2a6*,* Cyp2b13/Cyp2b9*,* Cyp4a11*,* Cyp4a14*,* Cyp4a22*,* Cyp7b1*,* Egr1*,* Elovl6*,* Gsta5*,* Gstp1*,* Hamp/Hamp2*,* Hsd3b4*,* Inhba*,* Keg1*,* Lpl*,* Mup1*,* Nat8*,* Ndrg1*,* Per2*,* Rorc*,* Scp2*,* Selenbp1*, and* Thrsp*) were identified by DNA chip analysis.* Rorc* and* Cyps* (cytochrome P450s) play key roles in Network 1. These analyses not only revealed the diverse spectrum of lipid nutrients regulated by* Cyps* but also clearly indicated that the balance of these mediators changes with dietary intake of different classes of polyunsaturated fatty acids. Network 2 functions in energy production, lipid metabolism, and small molecule biochemistry (Figure 4(b)) and includes 35 nodes (genes); 18 genes (*Aacs*,* Abcb11*,* Alas2*,* Csrp3*,* Cux2*,* CYPyp2a13/Cyp2a6*,* Cyp2d6*,* Cyp4a11*,* Cyp4a14*,* Ddx3y*,* Elovl6*,* Fmo5*,* Hsd3bB7*,* Mup1*,* Slc22a9*,* Thrsp*,* Tkt*, and* Vamp7*) were identified by DNA chip analysis. This network overlaps with the lipid metabolism and small molecule biochemistry signaling network.* Ppar-γ* and* Lep* play key roles in Network 2, but these genes were not significantly up- or downregulated in* Cmah*-null mice. Network 3 functions in hematological system development and tissue morphology inflammatory responses (Figure 4(c)) and includes 35 nodes; 16 genes (*Asl*,* C6*,* Creld2*,* Cyp7b1*,* Elovl6*,* Fbxo21*,* G0s2*,* Gck*,* HbaBA1/Hba2*,* Hlab*,* Ivns1abp*,* Ndrg1*,* Rgs16*,* Spon2*,* St6gal1*, and* Usp2*) were identified by DNA chip analysis.* Ikbkb*,* Il4*,* Il5*, and* Fos* play key roles in Network 3. Finally, Network 4 functions in energy production, small molecule biochemistry, and cardiac stenosis (Figure 4(d)) and includes 35 nodes; 13 genes (*Abcg8*,* Fmo3*,* Gnat1*,* Hsp90ab1*,* Hspa8*,* Hspb1*,* Hsph1*,* Igfbp2*,* Nt5e*,* Scara5*,* Serpina12*,* Sgk1*, and* Tfrc*) were identified by DNA chip analysis.* Tp53*,* Ahr*, and* NFκB* play key roles in Network 4.

The scores for Networks 1, 2, 3, and 4 were 56, 30, 26, and 19, respectively, and they are shown in Figure 4(e). Collectively,* Cmah*-regulated genes were found to be associated with biological processes that are critical for liver function, such as lipid metabolism, small molecule biochemistry, energy production, and tissue morphology inflammatory response signaling pathways. These data suggest that simultaneous impairment of multiple processes required for normal liver function could be responsible for the progression of metabolic disorders such as hyperglycemia and diabetes in* Cmah*-null mice.

### 3.5. Mitochondrial Morphology and Mitochondrial DNA Analyses in Liver from* Cmah*-Null and Control Mice

**Figure 5:**
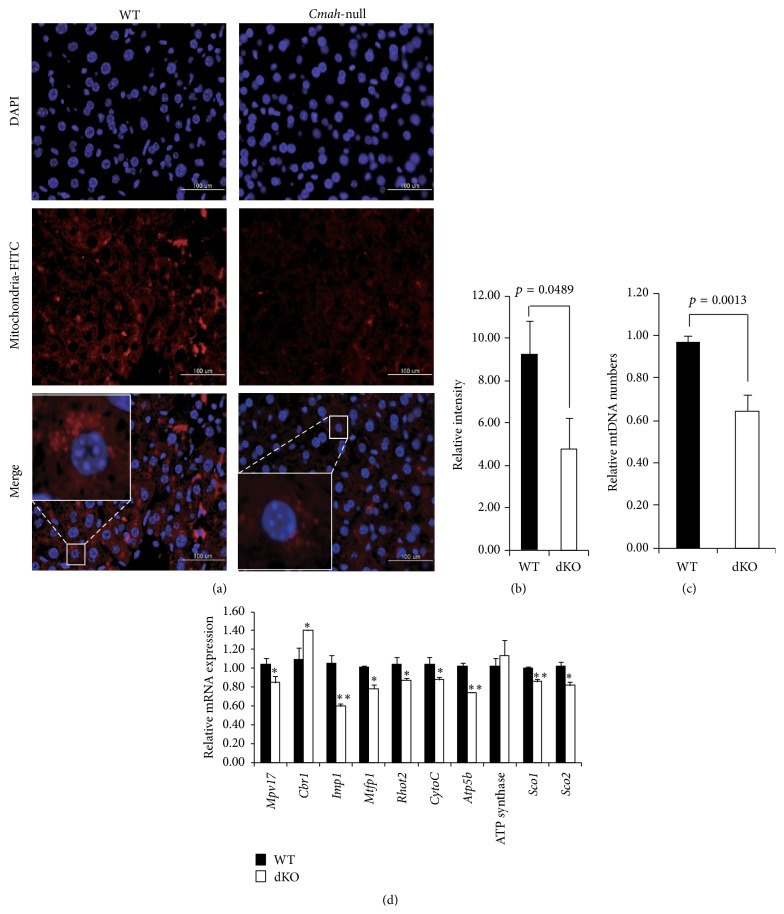
Characterization of mitochondrial dysfunction in liver of* Cmah*-null mice. (a) Reduction of mitochondrial activity in* Cmah*-null mice livers, stained using a mitochondrial antibody. (b) The intensity of the fluorescent signal indicates the level of mitochondrial activity. (c) The mtDNA/*β-actin* ratio, which represents the average copy number, was significantly decreased in* Cmah*-null mouse-derived livers. The* CytB* gene amplification level was normalized against the expression of nuclear *β-actin*. (d) Expression levels of genes involved in mitochondrial functional regulation were determined by RT-qPCR in RNA samples from the livers of WT and* Cmah*-null mice. Measurements were performed in triplicate, after which the mean expression was calculated and corrected using* gapdh* expression levels. Error bars indicate standard deviations. Significant differences are indicated by ^*∗*^ *p* < 0.05 and ^*∗∗*^ *p* < 0.01.

Immunohistochemical analysis of liver tissues from control and* Cmah*-null mice at 12 months of age showed variability in mitochondrial numbers in both groups but showed a significant decrease in positive signals for mitochondria in the liver of* Cmah*-null mice compared to control mice (Figures 5(a) and 5(b)). Next, we measured the quantity of mtDNA in livers of WT and* Cmah*-null mice by RT-qPCR (Figure 5(c)). The mtDNA/*β-actin* ratio, representing the average copy number, was significantly lower in* Cmah*-null mice (0.64 ± 0.079) than in WT (0.98 ± 0.033). Expression levels of genes related to mitochondrial activity and inheritance, such as genes required for subunits of ATP synthase (*Atp5b*), those essential for cytochrome-c oxidase function (*Soc1*,* Soc2*, and* CytoC*), those involved in mitochondrial trafficking (mitochondrial Rho GTPase 2,* Rhot2*), those required for mitochondrial energy production (mitochondrial inner membrane protein,* Mpv17*), those required for mitochondrial fission (mitochondrial fission process 1,* Mtfp1*), and those having a role in maintaining mitochondrial morphology and distribution (inner mitochondrial membrane peptidase-like,* Imp1*), were significantly downregulated (Figure 5(d)).

**Figure 6:**
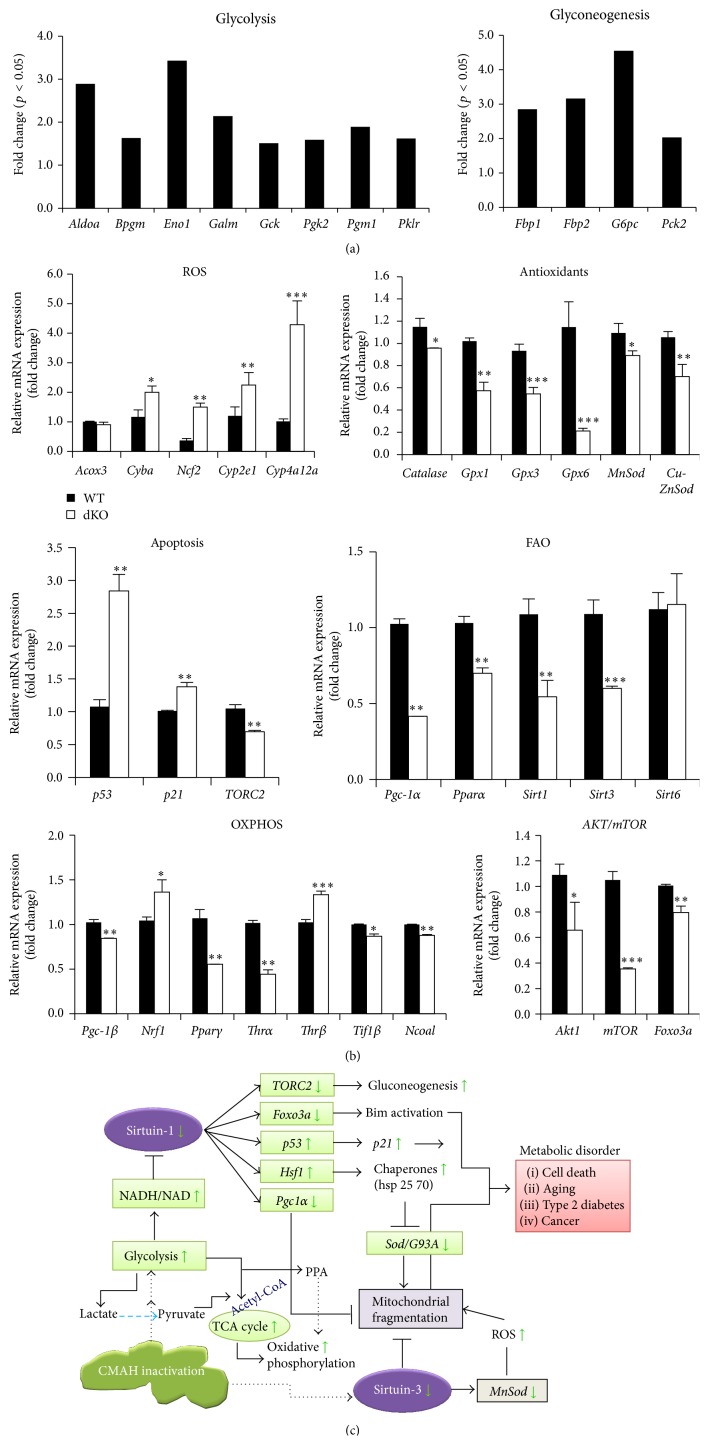
Molecular mechanisms of glucose-mediated regulation of sirtuins and oxidative stress in* Cmah*-null mice. (a) Expression of genes involved in glycolysis and glyconeogenesis, confirmed in WT and* Cmah*-null mouse-derived livers by a pathway-focused glucose metabolism PCR array. (b) Expression levels of genes involved in the molecular mechanisms of sirtuin and oxidative stress regulation were determined by RT-qPCR of RNA samples from liver tissues of WT and* Cmah*-null mice. Measurements were performed in triplicate, and the calculated mean expression was corrected using* Gapdh* expression levels. Error bars indicate standard deviations. Significant differences are indicated by ^*∗*^ *p* < 0.05 and ^*∗∗*^ *p* < 0.01, ^*∗∗∗*^ *p* < 0.001. (c) Glucose can directly or indirectly affect the main regulators of the aging process and sirtuin activity, as well as other contributors to aging such as oxidative stress. Increased glycolytic activity would tend to provoke an accumulation of NADH and lower the availability of NAD, resulting in decreased sirtuin activity. In addition, activation of the TCA cycle and *β*-oxidation of fatty acids distribute acetyl-CoA and acyl CoA, respectively, to the mitochondrial OXPHOS pathway. Therefore, lactate produced by glycolysis may be converted to pyruvate and ultimately enter the TCA cycle. Collectively, these results show that, via increased glycolysis, increased intracellular glucose levels can lead to (i) mitochondrial dysfunction and oxidative stress due to continuous ATP synthesis and (ii) accumulation of highly toxic advanced glycation end products (AGEs), which can provoke further oxidative stress. These observations suggest that the evolutionary loss of CMAH function may make humans more prone to diabetes or aging than other mammals.

To address the mechanisms underlying metabolic disorders in* Cmah*-null mice, we checked the expression profiles of genes whose expressions were significantly up- or downregulated by* Cmah* loss in the array analysis. As shown in Figure 6(a), we found that several major genes responsible for glycolysis and gluconeogenesis were significantly upregulated in mouse livers by* Cmah* loss. These findings were confirmed by a pathway-focused glucose metabolism PCR array. In this study, RT-qPCR analysis showed that expression of both of peroxisome proliferator-activated receptor gamma coactivator 1-alpha* (Pgc-1α)* and peroxisome proliferator-activated receptor gamma coactivator 1-beta* (Pgc-1β)* genes was lower in liver tissue of* Cmah*-null mice than in controls (Figure 6(b)). In addition, expression levels of* Sirt1*,* Pparα*, and* Pgc-1α* mRNAs, which are involved in fatty acid *β*-oxidation- (FAO-) related genes, were significantly lower in* Cmah*-null mice than in control mice (Figure 6(b)).* Sirt1* is a well-known positive regulator of both* Pparα* and* Pgc-1α*. Liver-specific decreased expression of* Sir1* mRNA may impair the hepatic response to fasting by inhibiting induction of* Pparα* target genes and may result in blunted FAO compared with controls. Collectively, these data suggest that the decreased mitochondrial activity observed in the livers of* Cmah*-null mice is primarily due to a decrease in the number, but not the mass, of mitochondria (Figure 6(c)).

To determine the regulatory factors that might control the expression of mitochondrial oxidative phosphorylation (OXPHOS) pathway genes in* Cmah*-null mouse-derived livers, we analyzed the expression of transcription factors and nuclear coactivators that are associated with OXPHOS or energy homeostasis, such as* Pgc-1α*,* Pgc-1β*, nuclear respiratory factor-1 (*Nrf1*), peroxisome proliferator-activated receptor (*Ppar*), thyroid hormone receptors (*Thrα*,* Thrβ*), steroid receptor coactivator-1 (*Ncoa1*), and transcriptional intermediary factor-1*β* (*Tif1β*). Gene expression levels of* Pgc-1β*,* Pparγ*,* Thrα*,* Ncoa1*, and* Tif1β*, but not of* Nrf1* or* Thrβ*, were significantly lower in* Cmah*-null mice than in controls (Figure 6(b)).

In accordance with the metabolic role of* Cmah*, mainly in glycogen and glucose accumulation, a number of genes involved in nutrient sensing, metabolism, and stress response were found. Sirtuins, which are involved in the regulation of various metabolic processes that allow the cell to adapt to nutrient stress, play a pivotal role in aging-related metabolic diseases [24]. This sirtuin activity modulates transcriptional regulation under oxidative stress. Therefore, we examined the changes in the expression of sirtuin and downstream oxidative stress genes regulated by glucose in* Cmah*-null mice. As shown in Figure 6(b),* Sirt1* and* Sirt3* expression were significantly decreased in* Cmah*-null mice, along with several antioxidant-related genes (*MnSod*,* Cu-ZnSod*,* Gpx1*,* Gpx3*,* Gpx6*, and* catalase*). This reduction in antioxidant gene expression could be due to the incapability of the cell to protect itself against oxidative stress, suggesting that downregulation of these genes can disrupt liver function in* Cmah*-null mice.

In addition, expression levels of mRNAs related to reactive oxygen species (ROS) generation (*Acox3*,* Cyba*,* Ncf2*,* Cyp2e1*, and* Cyp4a12a*) were significantly upregulated.* Cmah*-null livers showed significant upregulation of* p53* and* p21* mRNA expression due to downregulation of* Akt*,* mTOR*, and* Foxo3a* mRNA expression (Figure 6(b)). In conclusion, our findings suggest that* Cmah* inactivation may affect a number of signaling pathways by orchestrated changes in gene expression. The imbalance in redox status observed in the livers of* Cmah*-null mice indicates a nutritional oxidative stress phenomenon, which is caused by excessive and prolonged consumption of metabolic fuels (carbohydrates and lipids) and/or inadequate supply of dietary antioxidants. Based on our results, we propose a hypothetical model that explains how various signaling pathways could be affected by the loss of Neu5Gc in livers of* Cmah*-null mice (Figure 6(c)).

### 3.6. Validation of Microarray Results

A large number of genes that were identified as differentially regulated ones using DNA chip analysis were subjected to validation by RT-qPCR or western blot analysis, using* Gapdh* or *β-actin* as a reference gene or protein. We performed RT-qPCR to validate several randomly selected genes in the three biological groups shown in Table 2. As shown in Supplementary Figure  2(A), the mRNA levels of RT-qPCR precisely matched the microarray results. In addition, we performed a western blot analysis to investigate the relationship between the mRNA and protein expression levels. The protein expression levels of USP2 and GCK also matched the RT-qPCR data (Supplementary Figures  2(B) and 2(C)).

## 4. Discussion

Previous studies support the idea that CMAH plays a critical role in metabolic syndrome, that is, central obesity, carbohydrate intolerance, and type 2 diabetes mellitus [19]. However, the precise role of CMAH in the majority of these processes is still unclear. In the present study, we found that* Cmah*-null mice exhibited mitochondrial dysfunction due to oxidative stress. As a result, low levels of* Sirt1* and* Sirt3* gene expression, caused by high levels of NADH/NAD in* Cmah*-null mice, resulted in decreased* Foxo3a and MnSod* gene expression. The present study suggests that mitochondrial dysfunction caused by oxidative stress in* Cmah*-null mice may be either a cause or consequence of insulin resistance.

To address the possibility that in* Cmah*-null mice the liver generates excessive ROS, we checked the expression of genes involved in oxidative stress and redox imbalance. Genes encoding peroxisomal *β*-oxidation of fatty acids (*Acox3*), the nicotinamide adenine dinucleotide phosphate (*NADPH*) oxidase complex (*Cyba*,* Ncf2*,* Cyp2e1*, and* Cyp4a12a*), and stress-response genes (*Hspa8*,* Hspb1*,* Hsp90ab1*,* LOC666904*,* D3Ucla1*,* Hspa2*,* Ahsa1*, and* Hsp90ab1*), all of which are involved in ROS generation in the liver [25–27], were upregulated in* Cmah*-null mice (Figure 6 and Table 2). Expression levels of detoxification-related genes, such as* Gata1*,* Gstp1*,* Gsta3*,* Gsta2*,* and Gstm1*, were also significantly increased in* Cmah*-null mouse-derived livers (Table 2). In contrast, expression levels of antioxidant genes such as* Gpx1*,* Gpx3*,* Gpx6*,* MnSod*,* and Cu-ZnSod* were significantly downregulated (Figure 6(b)). Pathways related to carbohydrate metabolism (glycolysis, glycogen metabolism, other glycogen metabolisms, carbohydrate metabolism, monosaccharide metabolism, and other polysaccharide metabolisms) were also significantly upregulated in* Cmah*-null mouse-derived livers. Thus, increased glycolytic activity could lead to accumulation of NADH and eventually decreased activity of sirtuin. Therefore, increased intracellular glucose availability can lead to (i) mitochondrial dysfunction and oxidative stress as well as (ii) advanced glycation end products (AGEs) accumulation that can further induce oxidative stress. Finally, low* Sirt1* and* Sirt3* expression, due to high NADH/NAD, may decrease* Foxo-1* and* MnSod* expression, causing oxidative stress that results in cell death/aging or diabetes. These data provide the first molecular evidence that differential gene expression in* Cmah*-null mouse-derived livers regulates glucose metabolism in NADP-dependent and insulin-dependent manners due to CMAH inactivation.

It is well established that a reduced intake of dietary energy results in metabolic changes similar to those produced by fasting [28, 29]. The key changes include increased FAO and hepatic gluconeogenesis.* Pgc-1α* is a key regulator of glucose production in the liver of fasted and diabetic mice via activation of the entire gluconeogenic pathway [30–34]. The present study showed that, unlike fasting or reduced intake of dietary energy, loss of CMAH function was closely associated with decreased FAO-related gene expression, which is another major source of acetyl-CoA, such as* Sirt1*,* Sirt3*,* Pgc-1α*, and* Pparα* (Figure 6(b)). The results of the present study indicate that* Sirt1* and* Sirt3* appear to be nodes in a network designed to integrate metabolic signals and effectively control the production and utilization of energy via FAO and mitochondrial oxidative capacity. The exact mechanism by which CMAH regulates lipid metabolism is not known. One possibility is that CMAH directly inhibits a critical enzyme in the FAO pathway. In this study, loss of CMAH function resulted in decreased expression of FAO-related genes, which might contribute to CMAH loss-induced lipid accumulation.

OXPHOS can normally produce up to 38 mol ATP per mol glucose, depending on the efficiency of NADH shuttling into mitochondria as well as electron transport chain coupling, while glycolysis produces only 2 mol ATP per mol glucose [35]. In* Cmah*-null mice, the liver has a higher glycolytic flux and requires increased pentose phosphate pathways (PPP) for survival during oxidative stress, compared to controls (Figures 3(c), 3(d), and 3(e)). The group of genes related to glycolysis and tricarboxylic acid (TCA) cycle was the most significantly and coordinately altered group of genes in the livers of* Cmah*-null mice, compared to controls. The ratio of expression for many of the genes involved in the regulation of glycolysis and TCA cycle was significantly increased in* Cmah*-null mice (Figures 3(c) and 3(d)), compared to control mice, whereas FAO-related genes and regulatory factors controlling OXPHOS were significantly decreased (Figure 6(b)). In agreement with these findings, numbers of mitochondria were significantly decreased in the livers of* Cmah*-null mice (Figures 5(a) and 5(c)). Citrate synthase acts as a pace-making enzyme in the earliest step of the Krebs cycle, and isocitrate dehydrogenase catalyzes the third step of the cycle, that is, oxidative decarboxylation of isocitrate to form *α*-ketoglutarate and CO_2_ during the conversion of NAD+ to NADH [36]. In the present study, we found that expressions of citrate synthase and isocitrate dehydrogenase were significantly decreased in* Cmah*-null mouse-derived livers (Figures 3(c)–3(e)). In addition, we observed that expression of the* Atp5b* gene, related to ATP synthesis, was significantly downregulated in* Cmah*-null mouse-derived livers (Figure 5(d)). These decreases in gene expression indicated dysfunction in both the Krebs cycle enzymes and mitochondria. Collectively, certain mechanisms may limit energy production via OXPHOS in the livers of* Cmah*-null mice in terms of preference for glycolysis and its biosynthetic pathway branches, such as the PPP.

Next, we examined the expression of genes related to pathways in the livers of control and* Cmah*-null mice using a combination of Illumina MouseRef-8 v2 Expression BeadChip, a pathway-focused glucose metabolism PCR array, and microRNA PCR array analysis. We observed that lipid metabolism-, amino acid metabolism-, and mitochondrial respiratory function-related gene expressions were significantly dysregulated in* Cmah*-deficient livers, suggesting that a lack of CMAH function leads to perturbation of these metabolic pathways. In particular, expressions of lipid metabolism, small molecule biochemistry, inflammatory response, and cell death and survival genes were significantly increased in* Cmah*-null mouse-derived livers, leading to the development of metabolic syndrome, that is, central obesity, carbohydrate intolerance, type 2 diabetes mellitus, and dyslipidemia. As discussed above, these disorders may be caused by CMAH loss-induced lipid accumulation due to FAO dysregulation. Prior studies with* Cmah*-null mice showed that mice bearing a human-specific* Cmah* genetic mutation showed obesity-related metabolism and diabetes [18]. These data indicate that sialylation with Neu5Gc plays a significant role in the functioning of insulin-producing *β*-cells and its loss contributes to *β*-cell dysfunction in mice. Hedlund et al. [37] demonstrated that increased Neu5Gc expression is a feature of human cancers and it might be expected that neural tumors would express Neu5Gc. However, Neu5Gc is only found when tumor cells are injected subcutaneously into the flanks of mice and not when tumor cells are cultured independently, suggesting that the observed Neu5Gc was exogenous [38].

Collectively, these results suggest that* Cmah*-null mouse models mimic the human metabolic disorder phenotype and may enhance the scientific understanding of human metabolic disorders.

## 5. Conclusion

To identify consequences of the human-specific loss of Neu5Gc expression during evolution, we analyzed global gene expression profiles, gene interaction networks, and signaling pathways using an Illumina BeadChip microarray on liver tissues from WT and* Cmah*-null mice. By DNA chip analysis, we identified stress response, detoxification, and antioxidant genes that were up- and downregulated by the loss of CMAH activity. In addition, we also identified molecular pathways of sirtuins and oxidative stress that are regulated by glucose in* Cmah*-null mice. These findings suggest that enhanced glycolysis and the TCA cycle might contribute to the development of diabetes associated with metabolic syndrome. Further, the present study suggests that mice with CMAH deficiency can be taken as an important model for studying metabolic disorders in humans due to the evolutionary loss of Neu5Gc.

## Supplementary Material

2-2. Genotyping of Cmah-null mice; 2-3. Immunohistochemistry (IHC) and immunofluorescence staining; 2-8. RT-qPCR; 2-10. Western blot analysis.

## Figures and Tables

**Table 1:** KEGG pathway analysis of up- or downregulated genes in *Cmah-*null mouse-derived livers.

Pathway description	Number of genes	*p* value	Genes
Drug metabolism	16	5.34*E* − 10	*Cyp2d9, Gsta1, Gsta2, Gsta3, Cyp3a11, Cyp2b9, Cyp2c29, Cyp2b13, Adh7, Cyp2b10, Cyp2c50, Gstm1, Fmo5, Fmo3, Cyp2a5, *and* Gstp1*

Retinol metabolism	14	1.44*E* − 08	*Cyp3a11, Cyp2b9, Cyp2c29, Cyp2b13, Adh7, Cyp2b10, Cyp2c50, Cyp4a12b, Rdh11, Cyp4a12a, Dhrs4, Cyp4a31, Cyp2a5, *and* Cyp4a14*

Metabolism of xenobiotics by cytochrome P450	12	8.42*E* − 07	*Gstm1, Gsta1, Gsta2, Gsta3, Cyp2b9, Cyp3a11, Cyp2c29, Cyp2b13, Adh7, Cyp2b10, Gstp1, *and* Cyp2c50*

Arachidonic acid metabolism	10	2.83*E* − 04	*Cyp4a12b, Cbr1, Cyp4a12a, Cyp2b9, Cyp2c29, Cyp4a31, Cyp2b13, Cyp2b10, Cyp4a14, *and* Cyp2c50*

Steroid hormone biosynthesis	7	0.00101	*Cyp7b1, Cyp17a1, Hsd17b2, Cyp3a11, Hsd3b5, Srd5a1, *and* Hsd17b7*

Glutathione metabolism	7	0.00218	*Gstm1, Gsta1, Gsta2, Odc1, Gsta3, Srm, *and* Gstp1*

Insulin signaling pathway	10	0.00978	*G6pc, Irs2, Ppp1r3b, Gck, Pygl, Flot1, Pklr, Gys2, Mapk9, *and* Prkaa2*

Complement and coagulation cascades	7	0.01321	*C8b, C9, Serpinf2, Serpina5, C6, Serpina1e, *and* C1s*

Fatty acid metabolism	5	0.02916	*Cyp4a12b, Cyp4a12a, Cyp4a31, Adh7, *and* Cyp4a14*

**Table 2:** Classification of genes identified in *Cmah*-functional analysis.

Biological group	Pathway	Genes (fold change)
Protein metabolism and modification (*p* = 1.45*E* − 08)	Protein modification	*Prmt3* (1.57), *P4ha1* (1.53), *Ptpn11* (1.66), *Mapk9* (1.57), *1500003O03Rik* (1.53), *Man2a1* (1.71), *Siat9* (1.70), *St3gal5* (1.61), *St6gal1* (−2.02), *Trib3* (−1.93), *Ppm1k* (−1.56), *Sgk1* (−2.53), and *Plxna2* (−1.64)
	Protein folding	*Hsp105* (2.75), *Hspb1* (1.64), Tcp1 (1.63), *Dnajb1* (1.55), *Ero1lb* (1.71), *Hspa8* (3.15), *Hspb1* (2.11), *Hsp90ab1* (2.02), *LOC666904* (2.22), *D3Ucla1* (1.79), *Hspa2* (1.69), *Hsp90ab1* (1.53), *Fkbp5* (1.99), *Irak2* (1.55), *Camk2b* (1.66), *Bmx* (1.50), *Egfr* (1.75), and *Clpx* (−1.59)
	Proteolysis	*Usp2* (3.98), *Serpina12* (3.64), *Serpina5* (1.61), *Serpina1e* (1.57), *Rhbdl1* (1.56), *C1s* (1.52), *Ahsa1* (1.68), *Ppid* (1.63), *Bag3* (1.62), *Cul4a* (1.53), *Serpina7* (−1.66), *Serpinf2* (−1.65), *Usp18* (−1.62), *Itih2* (−1.61), *Ulk1* (−1.54), *Casp6* (−1.60), and *Mug2* (−1.56)
	Protein biosynthesis	*Mrps21* (1.59), *Eef2* (1.58), *Eif4b* (1.50), and *Eif4ebp3* (2.76)

Carbohydrate metabolism (*p* = 0.0105)	Glycolysis	*Gck* (2.10), *Pklr* (1.69)
	Glycogen metabolism	*Gys2* (1.92), *Ppp1r3b* (1.62), *Pygl* (1.54), and *G6pc* (−1.78)
	Other glycogen metabolisms	*Man2a1* (1.71), *Adh7* (1.67), and *Tkt* (2.14)
	Carbohydrate transport	*Slc35b1* (1.57)
	Monosaccharide metabolism	*Khk* (1.68), *Galt* (−1.53), and *Gnpda1* (−1.50)
	Other polysaccharide metabolisms	*St3gal5* (1.61), *Siat9* (1.70)

Immunity and defense (*p* = 1.83*E* − 07)	T-cell-mediated immunity	*LOC56628* (2.05), *H2-Q8* (1.72), *H2-Q10* (1.52), *Bmx* (1.50), *Raet1b* (−1.63), *Fkbp5* (1.99), *Cxcl1* (1.60), and *Gstt3* (−1.56)
	Macrophage-mediated immunity	*Cish* (1.92), *Sdc4* (−1.65)
	Complement-mediated immunity	*C6* (5.13), *C9* (1.87), *C8b* (1.53), *C1s* (1.52), and *Cfp* (−1.51)
	Stress response	*Hspa8* (3.15), *Hspb1* (2.11), *Hsp90ab1* (2.02), *LOC666904* (2.02), *D3Ucla1* (1.79), *Hspa2* (1.69), *Ahsa1* (1.68), and *Hsp90ab1* (1.53)
	Detoxification	*Gata1* (3.02), *Gstp1* (2.75), *Gsta3* (1.73), *Gsta2* (1.59), and *Gstm1* (1.55)

## References

[1] Irie A., Koyamat S., Kozutsumi Y., Kawasaki T., Suzuki A. (1998). The molecular basis for the absence of *N*-glycolylneuraminic acid in humans. *Journal of Biological Chemistry*.

[2] Hayakawa T., Satta Y., Gagneux P., Varki A., Takahata N. (2001). Alu-mediated inactivation of the human CMP-N-acetylneuraminic acid hydroxylase gene. *Proceedings of the National Academy of Sciences of the United States of America*.

[3] Brinkman-Van der Linden E. C. M., Sjoberg E. R., Juneja L. R., Crocker P. R., Varki N., Varki A. (2000). Loss of N-glycolylneuraminic acid in human evolution. Implications for sialic acid recognition by siglecs. *The Journal of Biological Chemistry*.

[4] Varki A. (2001). Loss of N-glycolylneuraminic acid in humans: mechanisms, consequences, and implications for hominid evolution. *American Journal of Physical Anthropology*.

[5] Deng L., Song J., Gao X. (2014). Host adaptation of a bacterial toxin from the human pathogen Salmonella Typhi. *Cell*.

[6] Higa H. H., Rogers G. N., Paulson J. C. (1985). Influenza virus hemagglutinins differentiate between receptor determinants bearing *N*-acetyl-, *N*-glycollyl-, and *N*,*O*-diacetyineuraminic acids. *Virology*.

[7] DeLuca G. M., Donnell M. E., Carrigan D. J., Blackall D. P. (1996). *Plasmodium falciparum* merozoite adhesion is mediated by sialic acid. *Biochemical and Biophysical Research Communications*.

[8] Klotz F. W., Orlandi P. A., Reuter G. (1992). Binding of *Plasmodium falciparum* 175-kilodalton erythrocyte binding antigen and invasion of murine erythrocytes requires *N*-acetylneuraminic acid but not its O-acetylated form. *Molecular and Biochemical Parasitology*.

[9] Reed M. B., Caruana S. R., Batchelor A. H., Thompson J. K., Crabb B. S., Cowman A. F. (2000). Targeted disruption of an erythrocyte binding antigen in *Plasmodium falciparum* is associated with a switch toward a sialic acid-independent pathway of invasion. *Proceedings of the National Academy of Sciences of the United States of America*.

[10] Martin M. J., Rayner J. C., Gagneux P., Barnwell J. W., Varki A. (2005). Evolution of human-chimpanzee differences in malaria susceptibility: relationship to human genetic loss of N-glycolylneuraminic acid. *Proceedings of the National Academy of Sciences of the United States of America*.

[11] Löfling J., Lyi S. M., Parrish C. R., Varki A. (2013). Canine and feline parvoviruses preferentially recognize the non-human cell surface sialic acid *N*-glycolylneuraminic acid. *Virology*.

[12] Springer S. A., Diaz S. L., Gagneux P. (2014). Parallel evolution of a self-signal: humans and new world monkeys independently lost the cell surface sugar Neu5Gc. *Immunogenetics*.

[13] Naito Y., Takematsu H., Koyama S. (2007). Germinal center marker GL7 probes activation-dependent repression of *N*-glycolylneuraminic acid, a sialic acid species involved in the negative modulation of B-cell activation. *Molecular and Cellular Biology*.

[14] Kwon D.-N., Lee K., Kang M.-J. (2013). Production of biallelic CMP-Neu5Ac hydroxylase knock-out pigs. *Scientific Reports*.

[15] Hedlund M., Tangvoranuntakul P., Takematsu H. (2007). N-glycolylneuraminic acid deficiency in mice: implications for human biology and evolution. *Molecular and Cellular Biology*.

[16] Chandrasekharan K., Yoon J. H., Xu Y. (2010). A human-specific deletion in mouse Cmah increases disease severity in the mdx model of Duchenne muscular dystrophy. *Science Translational Medicine*.

[17] Kwon D.-N., Chang B.-S., Kim J.-H. (2014). Gene expression and pathway analysis of effects of the CMAH deactivation on mouse lung, kidney and heart. *PLoS ONE*.

[18] Kavaler S., Morinaga H., Jih A. (2011). Pancreatic *β*-cell failure in obese mice with human-like CMP-Neu5Ac hydroxylase deficiency. *The FASEB Journal*.

[19] Kwon D. N., Chang B. .S., Kim J. H. (2014). MicroRNA dysregulation in liver and pancreas of CMP-Neu5Ac hydroxylase null mice disrupts insulin/PI3K-AKT signaling. *BioMed Research International*.

[20] Huang D. W., Sherman B. T., Lempicki R. A. (2009). Systematic and integrative analysis of large gene lists using DAVID bioinformatics resources. *Nature Protocols*.

[21] Gehring S., Dickson E. M., San Martin M. E. (2006). Kupffer cells abrogate cholestatic liver injury in mice. *Gastroenterology*.

[22] Winwood P. J., Arthur M. J. P. (1993). Kupffer cells: their activation and role in animal models of liver injury and human liver disease. *Seminars in Liver Disease*.

[23] Adeva M., González-Lucán M., Seco M., Donapetry C. (2013). Enzymes involved in l-lactate metabolism in humans. *Mitochondrion*.

[24] Li X., Kazgan N. (2011). Mammalian sirtuins and energy metabolism. *International Journal of Biological Sciences*.

[25] De Minicis S., Bataller R., Brenner D. A. (2006). NADPH oxidase in the liver: defensive, offensive, or fibrogenic?. *Gastroenterology*.

[26] Osmundsen H., Bremer J., Pedersen J. I. (1991). Metabolic aspects of peroxisomal beta-oxidation. *Biochimica et Biophysica Acta—Lipids and Lipid Metabolism*.

[27] Begriche K., Igoudjil A., Pessayre D., Fromenty B. (2006). Mitochondrial dysfunction in NASH: causes, consequences and possible means to prevent it. *Mitochondrion*.

[28] Adam-Perrot A., Clifton P., Brouns F. (2006). Low-carbohydrate diets: nutritional and physiological aspects. *Obesity Reviews*.

[29] Anson R. M., Guo Z., de Cabo R. (2003). Intermittent fasting dissociates beneficial effects of dietary restriction on glucose metabolism and neuronal resistance to injury from calorie intake. *Proceedings of the National Academy of Sciences of the United States of America*.

[30] Yoon J. C., Puigserver P., Chen G. (2001). Control of hepatic gluconeogenesis through the transcriptional coactivator PGC-1. *Nature*.

[31] Herzig S., Long F., Jhala U. S. (2001). CREB regulates hepatic gluconeogenesis through the coactivator PGC-1. *Nature*.

[32] Puigserver P., Rhee J., Donovan J. (2003). Insulin-regulated hepatic gluconeogenesis through FOXO1-PGC-1alpha interaction. *Nature*.

[33] Rhee J., Inoue Y., Yoon J. C. (2003). Regulation of hepatic fasting response by PPAR*γ* coactivator-1*α* (PGC-1): requirement for hepatocyte nuclear factor 4*α* in gluconeogenesis. *Proceedings of the National Academy of Sciences of the United States of America*.

[34] Lin J., Wu P.-H., Tarr P. T. (2004). Defects in adaptive energy metabolism with CNS-linked hyperactivity in PGC-1alpha null mice. *Cell*.

[35] Zhang J., Nuebel E., Daley G. Q., Koehler C. M., Teitell M. A. (2012). Metabolic regulation in pluripotent stem cells during reprogramming and self-renewal. *Cell Stem Cell*.

[36] Xue Y., Chen Q., Ding T., Sun J. (2014). SiO_2_ nanoparticle-induced impairment of mitochondrial energy metabolism in hepatocytes directly and through a Kupffer cell-mediated pathway in vitro. *International Journal of Nanomedicine*.

[37] Hedlund M., Padler-Karavani V., Varki N. M., Varki A. (2008). Evidence for a human-specific mechanism for diet and antibody-mediated inflammation in carcinoma progression. *Proceedings of the National Academy of Sciences of the United States of America*.

[38] Seyfried T. N., El-Abbadi M., Roy M. L. (1992). Ganglioside distribution in murine neural tumors. *Molecular and Chemical Neuropathology*.

