# Supplementary material for: CMP-Neu5Ac Hydroxylase Null Mice as a Model for Studying Metabolic Disorders Caused by the Evolutionary Loss of Neu5Gc in Humans
Source: Biomed Res Int. 2015 Oct 19;2015:830315. doi: 10.1155/2015/830315 (PMC4629002; doi:10.1155/2015/830315)
Supplement: Supplementary file 1 — 2-2. Genotyping of Cmah-null mice; 2-3. Immunohistochemistry (IHC) and immunofluorescence staining; 2-8. RT-qPCR; 2-10. Western blot analysis. [file 830315.f1.zip › Supplementary_data.doc]

***Supplementary information***

**CMP-Neu5Ac hydroxylase null mice as a model for studying metabolic disorders caused by evolutionary loss of Neu5Gc in human**

Deug-Nam Kwon, Yun-Jung Choi, Ssang-Goo Cho, Chankyu Park, Han Geuk Seo, Hyuk Song, and Jin-Hoi Kim*

Department of Animal Biotechnology, Konkuk University, Seoul 143-701, Republic of Korea

Supplementary data include 2 supplementary figures, 4 supplementary tables, and 4 additional files.

***To whom correspondence should be addressed:** Jin-Hoi Kim, PhD

Department of Animal Biotechnology, Konkuk University, 1 Hwayang-dong, Kwangjin-gu, Seoul 143-701, Republic of Korea

Telephone: +82-2-450-3687

Fax: +82-2-458-5414

E-mail: [jhkim541@konkuk.ac.kr](mailto:jhkim541@konkuk.ac.kr)

**Supplementary Figure 1. Confirmation of *Cmah-null* mice.** (A) Genotyping of *Cmah-null* mice. lane 1: wild type (WT) (+/+: 290 bp), lane 2: heterozygote (+/-: 290 and 380 bp), lane 3: homozygote (-/-: 380 bp). (B) Western blot analysis for CMAH and Neu5Gc in *Cmah-null* mouse-derived liver tissue. The housekeeping protein actin was used as a reference for normalization. (C) Immunohistochemical analysis of *Cmah-null* mouse-derived liver tissue for detection of expression of CMAH and Neu5GC. WT mice expressed both CMAH and Neu5Gc epitopes, but *Cmah-null* mice showed a complete deficiency of both CMAH and Neu5Gc epitopes in liver tissues. Bar: 100 μm.

**Supplementary Figure 2. Validation of gene expression by quantitative real-time RT-PCR and Western blot.** (A) Gene expression levels of selected genes were determined by quantitative real-time RT-PCR in RNA samples of liver of WT and *Cmah-null* mice. Measurements were performed in triplicate, after which the calculated mean expression was corrected for Gapdh expression levels. Error bars indicate standard deviations. *Usp2*: ubiquitin specific peptidase 2; *Serpina5*: serine (or cysteine) peptidase inhibitor, clade A (alpha-1 antiproteinase, antitrypsin), member 5; *Serpina1e*: serine (or cysteine) peptidase inhibitor, clade A, member 1e; *Serpina12*: serine (or cysteine) peptidase inhibitor, clade A (alpha-1 antiproteinase, antitrypsin), member 12; *Serpina7*: serine (or cysteine) peptidase inhibitor, clade A (alpha-1 antiproteinase, antitrypsin), member 7 for protein metabolism and modification related genes, *Fkbp5*: FK506 binding protein 5; *Raet1b*: retinoic acid early transcript beta; *C9*: complement component 9; *Cfp*: complement factor properdin for immunity and defense related genes; *Man2a1*: mannosidase 2, alpha 1; *GCK*: glucokinase; *Slc35b1*: solute carrier family 35, member B1; *Gys2*: glycogen synthase 2; *G6pc*: glucose 6 phosphatase, catalytic, 3; *Tkt*: transketolase for Carbohydrate metabolism related genes. (B) Western blot analysis for Usp2 (69 kda) and GCK (50 kda). The amount of total protein loaded per lane was 40μg; actin was analyzed as a loading control. (C) Relative intensity of differentially expressed proteins (Usp2 and GCK) in WT and *Cmah-null* by Western blotting.

**Supplementary Table 1. Source of antibodies used in this study**

| **Primary antibodies** | **Host** | **Dilution** | **Source** |
| --- | --- | --- | --- |
| CMAH | Mouse | 1:200 | Santa Cruz Biotechnology, CA, USA |
| Neu5Gc | Chicken | 1:1000 | Sialix, CA, USA |
| F4/80 | Rat | 1:100 | Abcam, Cambridge, UK |
| Mitochodria | Mouse | 1:100 | Abcam, Cambridge, UK |
| USP2 | Rabbit | 1:1000 | Proteintech, IL, USA |
| GCK | Rabbit | 1:1000 | Santa Cruz Biotechnology, CA, USA |
| Anti beta actin antibody | Rabbit | 1:2000 | Abcam, Cambridge, UK |
| **Secandary antibodies** | **Host** | **Dilution** | **Source** |
| Alexa Fluor 488 anti-rat IgG | Goat | 1:200 | Molecular Probes/Invitrogen, Carlsbad, CA |
| Alexa Fluor 488 anti-mouse IgG | Goat | 1:200 | Molecular Probes/Invitrogen, Carlsbad, CA |
| Anti-Chicken IgY (IgG) | Rabbit | 1:5000 | Sigma-aldrich |
| Goat anti-mouse IgG - HRP | Goat | 1:5000 | Santa Cruz Biotechnology, CA, USA |
| Donkey anti Rabbit IgG-HRP | Donkey | 1:5000 | Abcam, Cambridge, UK |

**Supplementary Table 2. Primer sets for mitochondrial DNA quantification and mitochondria functional regulation**

| **Genes** | **Forward primer** | **Reverse primer** | **Producr (bp)** |
| --- | --- | --- | --- |
| *mCytb* | ATTGACCTACCTGCCCCATC | CTCGTCCGACATGAAGGAAT | 247 |
| *mActinb* | TCGCCATGGATGACGATA | CACGATGGAGGGGAATACAG | 110 |
| *Mpv17* | TCGGAGGCTGGTACAAAGTT | ATTGTCCTGGGCTGACATTC | 161 |
| *Cbr1* | AGTGGTGAATGTGTCCAGCA | CAGGACTGTCACCCCAATCT | 211 |
| *IMP1* | GGCATCCAAAGAGGTGACAT | ACATGACCTGTTGGCACGTA | 155 |
| *Mtfp1* | GCTGTGGTGTGGTTGAGCTA | ACACAGACGGTTGATGGTGA | 198 |
| *Rhot1* | CAGTTACCCGCGAGAAGAAG | GGCTGTCAGCATTCACTTCA | 240 |
| *CytoC* | TTCCACAACCCTCATGTGAA | TAAGGGTCCAAAACCAGTGC | 109 |
| *ATP5b* | TGGGAAAATCGGACTCTTTG | AGTAACCACCATGGGCTTTG | 95 |
| *ATP synthesis* | GCCCTCGGTAATGCTATTGA | CACAGAGATTCGGGGGATAA | 135 |
| *Sco1* | AGTGGCCTTGAAAGAAAGCA | GTAGCCTGCCCTTGCTATTG | 79 |
| *Sco2* | CCTTCGCTGAACTTGTCCTC | CCCTAGAGCCAGTAGCATCG | 100 |

**Supplementary Table 3. Primer sets used for identify molecular mechanisms of sirtuins and oxidative stress regulation**

| **Genes** | **Forward primer** | **Reverse primer** | **Product (bp)** |
| --- | --- | --- | --- |
| *Acox3* | GCAAGAAACAACAGCCAGGT | TGTTCCCCAGAAATGTAGCC | 215 |
| *Cyba* | AAAGAGGAAAAAGGGGTCCA | TAGGCTCAATGGGAGTCCAC | 239 |
| *Ncf2* | GCAGTGGCCTACTTCCAGAG | CTTCATGTTGGTTGCCAATG | 243 |
| *Cyp2e1* | AGGCTGTCAAGGAGGTGCTA | GGAAGTGTGCCTCTCTTTGG | 210 |
| *Cyp4a12a* | CAAGACAGCCCAGCTCTACC | CCCCAGAATCAGCTTCATGT | 229 |
| *Catalase* | CCCCCAACTATTACCCCAAC | TGTTCTCACACAGGCGTTTC | 186 |
| *Gpx1* | GTCCACCGTGTATGCCTTCT | TCTGCAGATCGTTCATCTCG | 152 |
| *Gpx3* | ACCAATTTGGCAAACAGGAG | AGCGGATGTCATGGATCTTC | 237 |
| *Gpx6* | AAGGTGGATTGCAACAAAGG | CAAAACCGTGACGTTGAATG | 207 |
| *MnSOD* | GGCCAAGGGAGATGTTACAA | CCTTGGACTCCCACAGACAT | 213 |
| *Cu-ZnSOD* | CCAGTGCAGGACCTCATTTT | TTGTTTCTCATGGACCACCA | 197 |
| *p53* | AGAGACCGCCGTACAGAAGA | CTGTAGCATGGGCATCCTTT | 232 |
| *p21* | CGGTGGAACTTTGACTTCGT | CAGGGCAGAGGAAGTACTGG | 159 |
| *TORC2* | CACCCACCCCAAAGTCTCTA | GTTGATGGGCTCTCCATGTT | 205 |
| *PGC-1α* | AAGGTCCCCAGGCAGTAGAT | GCGGTATTCATCCCTCTTGA | 190 |
| *PPARα* | AGAAGTTGCAGGAGGGGATT | TCGGACTCGGTCTTCTTGAT | 166 |
| *Sirt1* | AAGGAGCAGATTAGTAAGC | GCACCGTGGAATATGTAA | 77 |
| *Sirt3* | TACAGGCCCAATGTCACTCA | ACAGACCGTGCATGTAGCTG | 167 |
| *Sirt6* | CTGGTCTGGAACTCACTGCT | CGGGTGTGATTGGTAGAGAG | 238 |
| *PGC-1β* | TTGTAGAGTGCCAGGTGCTG | GATGAGGGAAGGGACTCCTC | 220 |
| *NRF1* | GTTGGTACAGGGGCAACAGT | TCGTCTGGATGGTCATTTCA | 154 |
| *PPARγ* | TTTTCAAGGGTGCCAGTTTC | AATCCTTGGCCCTCTGAGAT | 198 |
| *Thrα* | GGCTGTGCTGCTAATGTCAA | CGGAGGTCAGTCACCTTCAT | 165 |
| *Thrβ* | GGGCTAGCCTGTGTTGAGAG | TAAGAACAGAGGCGGGAAGA | 216 |
| *TIF1β* | ACCTGACCTTGATTCGTGCT | TGGTTCTACCAGCACAGCAG | 221 |
| *Ncoa1* | TGGGTACCAGTCACCAGACA | GAATGTTTGCGTTTCCACCT | 161 |
| *AKT1* | CCCTTCTACAACCAGGACCA | ATACACATCCTGCCACACGA | 210 |
| *mTOR* | ACACAGTAATCCTTCAGA | GCAATGTTTATGATGAGTTT | 111 |
| *Foxo3a* | GGGGAGTTTGGTCAATCAGA | GCCTGAGAGAGAGTCCGAGA | 193 |

**Supplementary Table 4. Primer sets for quantitative real-time RT-PCR**

| Group | Genes | Forward primer | Reverse primer | Product (bp) |
| --- | --- | --- | --- | --- |
| Protein modification | *Usp2* | CTTCTGGGATCTCTCGTTGC | TGTTGTGAGCTTGCTGGTTC | 241 |
|  | *Serpina5* | CTGGCTTGAAGACGAAGACC | CTGGCTTGAAGACGAAGACC | 183 |
|  | *Serpina1e* | TTCCAACACCTCCTCCAAAC | CACTTTCTTGGCCTCCTCTG | 183 |
|  | *Serpina12* | GATTCTCCAGTCAGGGTCCA | AGGCAGTGGAGATGCTCAGT | 157 |
|  | *Serpina7* | CTCAGTGGACAAGAGCACCA | TGTGTCCCTCTTTTGGAAGG | 155 |
| Immunity and defense | *Fkbp5* | CAAAGCCTCAGAGTCGTTCC | GGATTGACTGCCAACACCTT | 219 |
|  | *Raet1b* | GAGACAGCAAATGCCACTGA | TTGCGGATAAATCATGGTGA | 153 |
|  | *C9* | AAGGTTTCGCTCAAGAAGCA | TCCACAGTCGTTGTCACCAT | 199 |
|  | *Cfp* | GACATGTGATCACCCTGCAC | TGGCTTCCCATTAAATTTGC | 229 |
| Carbohydrate metabolism | *Man2a1* | TTCGTCCTTCCTCAACCATC | CCTTTCCTGTGGAGGATCAA | 201 |
|  | *Gck* | CACATGTGCTCAGCAGGACT | TGAAGGTGATTTCGCAGTTG | 178 |
|  | *Slc35b1* | TAAATCCTGCAAGCCAATCC | CCTGGGAAACACCTGTCAGT | 224 |
|  | *Gys2* | GGGACACTGTGCATTGTTTG | CCGATTCGTCTAATGGTGCT | 223 |
|  | *G6pc* | TCTGTCCCGGATCTACCTTG | GTAGAATCCAAGCGCGAAAC | 172 |
|  | *Tkt* | GCCCTTCTGCAGTACTTTCG | GCCTTCTCTGTTGCAACTCC | 222 |

**Additional files**

Additional file 1. Up-regulated genes in *Cmah-null* mice (xls file)

Additional file 2. Down-regulated genes in *Cmah-null* mice (xls file)

Additional file 3. 31 Biological process in up-regulated genes (xls file)

Additional file 4. 31 Biological process in down-regulated genes (xls file)
